# 25012 Expanding Community Knowledge and Relationships for Congregation-Neighbor Health Connections and Advocacy in Indianapolis through a #HealthyMe Learning Community

**DOI:** 10.1017/cts.2021.608

**Published:** 2021-03-30

**Authors:** David Craig, Shonda Gladden, Jacob Christenson, Dustin Lynch, Meredith Campbell, Emily Hardwick, Sarah Wiehe

**Affiliations:** 1 IUPUI Liberal Arts - Religious Studies; 2 Good to the Soul; 3 IU School of Medicine - Indiana Clinical & Translational Sciences Institute; 4 IU School of Medicine

## Abstract

ABSTRACT IMPACT: Congregations’ support for social, emotional, mental and spiritual wellness is foundational to human health and their community knowledge and presence can improve resilience and health in socially vulnerable neighborhoods. OBJECTIVES/GOALS: The Indiana CTSI Monon Collaborative is listening and understanding the most pressing health issues in the community and are working together to design and deliver community health solutions. We worked with our community ambassador to launch a health and wellness learning community for ten congregations seeking to build a health-connector network. METHODS/STUDY POPULATION: Study team used qualitative (interviews, focus groups, listening sessions, learning management system, participatory-design research) and quantitative (surveys) data collection methods in the development and ongoing implementation of the learning community. Study Population: Based on initial assessment of health and social vulnerability data within the Marion County neighborhoods in Indianapolis, community ambassador engaged congregations in more vulnerable neighborhoods to seek participation in learning community. Ten congregations signed a covenant of participation; learning community includes 10 clergy and 8 health advocates. RESULTS/ANTICIPATED RESULTS: Since the inception of the Learning Community in May 2020, we have developed a better understanding of the assets and barriers of LC participants around health and well-being. Through ongoing virtual gatherings (facilitated by community ambassador Good to the Soul), sharing of resources through our online modules on Canvas (LMS), and synthesis of data captured throughout our time together, LC participants have developed SMART goals which will inform priority setting for congregations to assist them in identifying the resources and connections necessary to drive forward solutions together as they seek out funding opportunities to support health improvement. DISCUSSION/SIGNIFICANCE OF FINDINGS: The learning community has provided a space and structure for congregations to align around a shared goal focused on health and wellness. Through regular gatherings we were able to connect people, organizations, and systems who were all eager to learn and work across boundaries leading to greater resilience in vulnerable communities.

